# Pamphlets Received

**Published:** 1854-07

**Authors:** 


### *Pamphlets Received.*

*Remarks on Croup and its Treatment.* By HORACE GREEN, M. D.

This paper, (originally published in the American Medical Monthly,) reviews the various methods of treatment adopted in Croup, and advocates strongly the topical treatment by the nitrate of silver. The weight of testimony is now largely in favor of the caustic, and it is becoming very generally adopted, and, so far as we can learn, with an increasing success.

Dr. Green's paper is a concise and able argument.

*A Monograph on the Fœtal Circulation.* By E. R. PEASLEE, A. M., M. D., Prof. of Anatomy in Dartmouth College and in the New York Medical College, and of Surgery in the Medical School of Maine.

This, like Dr. Green's paper, was originally published in the American Medical Monthly. We regret that we have not room for the conclusions at which it arrives. They are, to a considerable extent, original, and are worthy of the attention of the physiologist.

*Report to Louisiana State Medical Society on the Meteorology, Vital Statistics and Hygiene of the State of Louisiana.* By E. H. BARTON, M. D., Prof. of Theory and Practice of Medicine in the University of La., etc.

Dr. Barton is widely known as a thorough, persevering, and accomplished scholar in epidemiological study. In this report, (one of the most valuable in our knowledge,) he gives to meteorology that importance which it deserves as a cause of disease.

Dr. Barton's records of the hygrometrical condition of the atmosphere extend through a period of eight years, with three to five observations daily. Such an amount of labor must needs bring forth valuable results. Dr. B. will please accept our thanks for his kind compliance with our request.

*Strictures on Professor Espy's Theory of Storms.* By ROBERT HARE, M. D.

Prof. Hare does not agree to Prof. Espy's theory of upward radiation of heat as a cause of storms. He deems the cause insufficient for the effect, and offers a beautiful electrical theory, which we must condense into so brief a space that only its general outlines can appear.

Electrical discharges may occur either from the clouds to the earth, or *vice versa.* These discharges are of two kinds: convective and diruptive.

The diruptive discharge is accompanied by a flash and report; the convective is silent, more diffused, and less concentrated than the diruptive. An upward convective discharge of electricity would produce an upward current of air, having a vacuum to be filled by surrounding atmosphere. The extent, force, and suddenness of such a discharge will determine the character of the wind, whether a breeze merely, or a hurricane or tornado.

Such a theory certainly harmonizes with the phenomena of tornadoes, better than mere radiation of heat. It is, however, very evident that the unequal cooling of large bodies of land and water also materially influences the force and direction of winds.

*Eleventh Annual Report of the Managers of the State Lunatic Asylum of the State of New York.*

The number of patients at the commencement of the year was: males, 115; females, 210; total, 425. Admitted during the year: males, 251; females, 173; total, 424. Whole number treated: males, 466; females, 383; total, 849. Discharged: males, 227; females, 176; total, 404. Remaining, Nov. 30th, 1843: males, 239; females, 207; total, 446.

*Proceedings of the First Meeting of the General Committee appointed by the World's Temperance Convention, with their Address, &c.*

*Seventeenth Annual Report of the Superintendent of Public Schools of the City of Buffalo—for* 1853.

This report proposes a system of hygienic teaching in our schools. The plan is yet in embryo, but we trust that it may be carried out in full. Few things go farther to make the good and orderly citizen, than the inculcation of habits of neatness, and an intelligent regard for the interests of health. Vice is not exactly a matter of dirt and bad ventilation, but cleanliness, good food, and pure air, have much to do in guiding the disposition of the individual.

*Seventh Annual Report of the Regents of the University on the Condition of the State Cabinet of Natural History.*

